# Do countries rely on the World Health Organization for translating research findings into clinical guidelines? A case study

**DOI:** 10.1186/s12992-016-0196-2

**Published:** 2016-10-06

**Authors:** Ramadhani A. Noor, Pascal Geldsetzer, Till Bärnighausen, Wafaie Fawzi

**Affiliations:** 1 Department of Global Health and Population, Harvard T.H. Chan School of Public Health, 677 Huntington Avenue, Boston, MA02115 USA; 2 Department of Nutrition, Harvard T.H. Chan School of Public Health, 677 Huntington Avenue, Boston, MA02115 USA; 3 Africa Academy for Public Health (AAPH), Plot #802, Mwai Kibaki Road, Dar es Salaam, Tanzania; 4 Africa Health Research Institute, Mtubatuba, 3935 South Africa; 5 Institute of Public Health, Heidelberg University, 69120 Heidelberg, Germany; 6 Department of Epidemiology, Harvard T.H. Chan School of Public Health, 677 Huntington Avenue, Boston, MA02115 USA

**Keywords:** World Health Organization, Guidelines, HIV, Nutrition, Micronutrient supplementation

## Abstract

**Background:**

The World Health Organization’s (WHO) antiretroviral therapy (ART) guidelines have generally been adopted rapidly and with high fidelity by countries in sub-Saharan Africa. Thus far, however, WHO has not published specific guidance on nutritional care and support for (non-pregnant) adults living with HIV despite a solid evidence base for some interventions. This offers an opportunity for a case study on whether national clinical guidelines in sub-Saharan Africa provide concrete recommendations in the face of limited guidance by WHO. This study, therefore, aims to determine if national HIV treatment guidelines in sub-Saharan Africa contain specific guidance on nutritional care and support for non-pregnant adults living with HIV.

**Methods:**

We identified the most recent national HIV treatment guidelines in sub-Saharan African countries with English as an official language. Using pre-specified criteria, we determined for each guideline whether it provides guidance to clinicians on each of five components of nutritional care and support for adults living with HIV: assessment of nutritional status, dietary counseling, micronutrient supplementation, ready-to-use therapeutic or supplementary foods, and food subsidies.

**Results:**

We found that national HIV treatment guidelines in sub-Saharan Africa generally do not contain concrete recommendations on nutritional care and support for non-pregnant adults living with HIV.

**Conclusions:**

Given that decisions on nutritional care and support are inevitably being made at the clinician-patient level, and that clinicians have a relative disadvantage in systematically identifying, summarizing, and weighing up research evidence compared to WHO and national governments, there is a need for more specific clinical guidance. In our view, such guidance should at a minimum recommend daily micronutrient supplements for adults living with HIV who are in pre-ART stages, regular dietary counseling, periodic assessment of anthropometric status, and additional nutritional management of undernourished patients. More broadly, our findings suggest that countries in sub-Saharan Africa look to WHO for guidance in translating evidence into clinical guidelines. It is, thus, likely that the development of concrete recommendations by WHO on nutritional interventions for people living with HIV would lead to more specific guidelines at the country-level and, ultimately, better clinical decisions and treatment outcomes.

## Background

### The World Health Organization’s HIV treatment guidelines

Over the last two decades, the World Health Organization (WHO) has periodically published new HIV care and treatment guidelines. Its latest guidelines, published in 2016, are 480 pages in length and cover a variety of topics related to HIV care and treatment, including clinical guidance on HIV diagnosis, eligibility for antiretroviral therapy (ART), the clinical monitoring of patients, and treatment of co-morbidities. Additional guidance is provided on service delivery topics, such as linkage from HIV testing to care, HIV care retention, and interventions to increase ART adherence [1].

Much of the guidance provided in the WHO HIV guidelines is specific; for example, they include precise clinical criteria to determine when to initiate ART, which, until 2016, was mostly based on certain CD4-cell count thresholds and clinical staging, as well as the names of antiretroviral drugs to be used as first- and second-line ART regimens [1, 2]. The WHO has reported that most countries adopted the sections on ART eligibility and antiretroviral drug regimens from its 2010 ART guidelines within two years of publication [3]; the adoption of the WHO 2013 guidelines was even more rapid [3]. While it seems to be the case that governments closely follow the WHO’s guidelines on ART eligibility and antiretroviral drug regimens [3], there is, to the best of our knowledge, no published evidence on whether governments issue specific guidelines to their clinicians and public health program managers on topics in HIV care and treatment, for which the WHO has *not* provided any specific recommendations. If national HIV treatment guidelines follow the lack of specific guidance on certain questions provided by the WHO guidelines, then this would present, in our view, a clearly dissatisfying state of affairs. After all, compared to the WHO or Ministries of Health, clinicians generally 1) are more time constrained, and less trained, to gather and interpret existing evidence (e.g., through a systematic review and meta-analysis), and 2) have less access to subject-specific and methodological expertise.

### The example of nutritional care and support for adults living with HIV

The WHO has published guidelines on nutritional care and support for pregnant women, HIV-exposed infants, and children living with HIV [2, 4–6]. Additionally, it conducted a technical consultation on nutrient requirements for people living with HIV in 2003 [7]. However, the WHO has not published any guidelines on nutritional care and support for non-pregnant adults living with HIV other than one paragraph, which is identical in the WHO’s two latest ART guidelines. The WHO writes in both its 2013 and 2016 ART guidelines:

*“Nutritional assessment (anthropometry, clinical and dietary assessment), counselling and support should be an integral component of HIV care and conducted at enrolment in care and monitored during all HIV care and treatment. Malnourished HIV patients, especially in food insecure contexts, may require food supplements, in addition to ART, to ensure appropriate foods are consumed to support nutritional recovery. Weight loss or failure to regain or maintain a healthy weight at any stage of HIV infection or ART should trigger further assessment and appropriate interventions.” (WHO 2013, p. 172; and WHO 2016, p. 222).*

While this paragraph provides guidance on the need for nutritional assessments, its components, and when it should first be conducted, it does not provide guidance on other key topics within nutritional care and support, such as micronutrient supplementation and ready-to-use therapeutic or supplementary foods. We review the evidence on these interventions for non-pregnant adults living with HIV (regardless of whether they are on ART or not) in the first part of the discussion section, showing that several interventions have a solid evidence base. Nutritional care and support for adults living with HIV, thus, presents an opportunity for a case study on whether individual governments issue specific guidance to their clinicians in the face of lacking guidelines by the WHO, or whether they instead fail to provide such guidance; thus, leaving the decision (explicitly or implicitly) to individual clinicians and program managers. By focusing on countries in sub-Saharan Africa, the region that is home to approximately 70 % of those living with HIV globally [8], we aim to determine whether national HIV treatment guidelines provide specific guidance to clinicians and program managers on nutritional care and support for non-pregnant adults living with HIV.

## Methods

We searched the AIDS Support and Technical Assistance Resources (AIDSTAR-One) database [9] and the Google search engine for the most recent 1) national HIV treatment guidelines, and 2) national nutritional guidelines for people living with HIV. National HIV treatment guidelines were defined as a country’s clinical guideline providing information on CD4-cell count thresholds for ART eligibility and ART regimens to be used by clinicians. National nutritional guidelines were included in this review if they were specifically published to provide guidance on nutritional care and support for people living with HIV; general nutritional care guidelines (e.g., guidelines on managing acute malnutrition) were excluded. For feasibility reasons, we restricted the search to countries in sub-Saharan Africa with English as one official language [10] to increase the likelihood that an English version of the guideline exists.

The search was conducted from September 26^th^, 2015, to October 4^th^, 2015. Prior to the search, we established criteria (listed in Tables 1 and 2) for specificity of the guidance in each of five components of nutritional care and support for adults living with HIV: 1) assessment of nutritional status, 2) dietary counseling, 3) micronutrient supplementation, 4) ready-to-use therapeutic or supplementary foods (RUTFs), and 5) food subsidies. The specificity criteria under each component were devised based on our own judgment of the information that a clinician would need to provide the relevant care to a patient. As such, we also recorded all instances, in which a guideline stated that a certain intervention (e.g., a micronutrient supplement) or service (e.g., dietary counseling) should not be provided.

## Results

National HIV treatment guidelines were identified for 18 of the 24 countries in sub-Saharan Africa, in which English is an official language. More specifically, we identified national HIV treatment guidelines for Botswana [11], Ethiopia [12], Ghana [13], Kenya [14, 15], Lesotho [16], Liberia [17], Malawi [18], Namibia [19], Nigeria [20], Rwanda [21], Sierra Leone [22], South Africa [23], South Sudan [24], Swaziland [25], Tanzania [26], Uganda [27], Zambia [28], and Zimbabwe [29]. In addition, we identified guidelines for nutritional care and support of individuals living with HIV for the following countries: Botswana [30], Kenya [31], Rwanda [32], South Africa [33], Swaziland [34], Tanzania [35], Uganda [36], and Zambia [37].

Except for recommending that adults living with HIV receive an assessment of nutritional status and dietary counseling at least once, the majority of HIV treatment guidelines did not provide specific guidance in the other three components of nutritional care and support, nor recommendations on when, how often, and how nutritional assessment and counseling should be conducted (Table 1). In particular, very few countries provided guidance on micronutrient supplementation and RUTFs, and none made recommendations on food subsidies (e.g., the provision of food baskets). The identified guidelines on nutritional care and support for people living with HIV tended to be only marginally more specific in their recommendations on nutritional care and support than the HIV treatment guidelines (Table 2), with the majority not making specific recommendations on micronutrient supplements, RUTFs, or food subsidies.

**Table 1:** Recommendations by national HIV treatment guidelines^a^ in five areas of nutritional care and support for non-pregnant HIV-infected adults

Component of nutritional care and support	Does the guideline specify…	Countries providing guidance	Countries *not* providing guidance
*Assessment of nutritional status*	… that an assessment of nutritional status should be carried out at least once?	Botswana, Ghana, Kenya, Lesotho, Liberia, Malawi, Namibia, South Sudan, Swaziland, Tanzania, Uganda	Ethiopia, Nigeria, Rwanda, Sierra Leone, South Africa, Zambia, Zimbabwe
	… when in the HIV care cascade nutritional status should first be assessed?	Kenya, Lesotho, Liberia, Malawi, South Sudan, Uganda	Botswana, Ethiopia, Ghana, Namibia, Nigeria, Rwanda, Sierra Leone, South Africa, Swaziland, Tanzania, Zambia, Zimbabwe
	… how regularly nutritional status should be re-assessed?	Kenya, Liberia, Malawi, South Sudan, Uganda	Botswana, Ethiopia, Ghana, Lesotho, Namibia, Nigeria, Rwanda, Sierra Leone, South Africa, Swaziland, Tanzania, Zambia, Zimbabwe
	… how the assessment should be carried out?	Botswana, Kenya, Liberia, Malawi, Namibia, South Sudan, Tanzania	Ethiopia, Ghana, Lesotho, Nigeria, Rwanda, Sierra Leone, South Africa, Swaziland, Uganda, Zambia, Zimbabwe
*Dietary counseling*	… that dietary counseling should be provided?	Botswana, Ghana, Kenya, Lesotho, Liberia, Malawi, Namibia, Nigeria, Sierra Leone, South Sudan, Tanzania, Uganda, Zimbabwe	Ethiopia, Rwanda, South Africa, Swaziland, Zambia
	… when in the HIV care cascade it should first be provided?	Malawi, Nigeria, South Sudan, Uganda	Botswana, Ethiopia, Ghana, Kenya, Lesotho, Liberia, Namibia, Rwanda, Sierra Leone, South Africa, Swaziland, Tanzania, Zambia, Zimbabwe
	… how regularly it should be provided?	Botswana, Malawi, South Sudan, Zimbabwe	Ethiopia, Ghana, Kenya, Lesotho, Liberia, Namibia, Nigeria, Rwanda, Sierra Leone, South Africa, Swaziland, Tanzania, Uganda, Zambia
	… what topics should be covered during the counseling?	Botswana, Kenya, Liberia, Namibia, Nigeria, Sierra Leone, Tanzania, Uganda	Ethiopia, Ghana, Lesotho, Malawi, Rwanda, South Africa, South Sudan, Swaziland, Zambia, Zimbabwe
*Micronutrient supplementation*	… whether a micronutrient supplement should be provided?	Kenya, Nigeria, South Sudan	Botswana, Ethiopia, Ghana, Lesotho, Liberia, Malawi, Namibia, Rwanda, Sierra Leone, South Africa, Swaziland, Tanzania, Uganda, Zambia, Zimbabwe
	… under what circumstances a micronutrient supplement should be provided?	Botswana, Kenya, South Sudan	Ethiopia, Ghana, Lesotho, Liberia, Malawi, Namibia, Nigeria, Rwanda, Sierra Leone, South Africa, Swaziland, Tanzania, Uganda, Zambia, Zimbabwe
	… which micronutrient(s) should be provided?	Botswana, Kenya	Ethiopia, Ghana, Lesotho, Liberia, Malawi, Namibia, Nigeria, Rwanda, Sierra Leone, South Africa, South Sudan, Swaziland, Tanzania, Uganda, Zambia, Zimbabwe
	… the dosage of at least one micronutrient supplement?	Kenya, South Sudan	Botswana, Ethiopia, Ghana, Lesotho, Liberia, Malawi, Namibia, Nigeria, Rwanda, Sierra Leone, South Africa, Swaziland, Tanzania, Uganda, Zambia, Zimbabwe
*Ready-to-use therapeutic and supplementary foods (RUTFs)*	… whether a RUTF should be provided?	Kenya, South Sudan, Tanzania, Uganda	Botswana, Ethiopia, Ghana, Lesotho, Liberia, Malawi, Namibia, Nigeria, Rwanda, Sierra Leone, South Africa, Swaziland, Zambia, Zimbabwe
	… under what circumstances a RUTF should be provided?	Kenya, Nigeria, South Sudan, Tanzania, Uganda	Botswana, Ethiopia, Ghana, Lesotho, Liberia, Malawi, Namibia, Rwanda, Sierra Leone, South Africa, Swaziland, Zambia, Zimbabwe
	… which RUTF(s) should be provided?	-	Botswana, Ethiopia, Ghana, Kenya, Lesotho, Liberia, Malawi, Namibia, Nigeria, Rwanda, Sierra Leone, South Africa, South Sudan, Swaziland, Tanzania, Uganda, Zambia, Zimbabwe
	… the dosage of at least one RUTF?	Kenya	Botswana, Ethiopia, Ghana, Lesotho, Liberia, Malawi, Namibia, Nigeria, Rwanda, Sierra Leone, South Africa, South Sudan, Swaziland, Tanzania, Uganda, Zambia, Zimbabwe
*Food subsidies*	… whether a food subsidy should be provided?	-	Botswana, Ethiopia, Ghana, Kenya, Lesotho, Liberia, Malawi, Namibia, Nigeria, Rwanda, Sierra Leone, South Africa, South Sudan, Swaziland, Tanzania, Uganda, Zambia, Zimbabwe
	… under what circumstances a food subsidy should be provided?	-	Botswana, Ethiopia, Ghana, Kenya, Lesotho, Liberia, Malawi, Namibia, Nigeria, Rwanda, Sierra Leone, South Africa, South Sudan, Swaziland, Tanzania, Uganda, Zambia, Zimbabwe
	… how the food subsidy should be provided (e.g., vouchers, food baskets, etc.)?	-	Botswana, Ethiopia, Ghana, Kenya, Lesotho, Liberia, Malawi, Namibia, Nigeria, Rwanda, Sierra Leone, South Africa, South Sudan, Swaziland, Tanzania, Uganda, Zambia, Zimbabwe

*Abbreviations*: *HIV* human immunodeficiency virus, *RUTF* Ready-to-use therapeutic or supplementary food

^a^National HIV treatment guidelines in English were not identified for the following countries: Cameroon, Eritrea, Mauritius, Seychelles, Sudan, and The Gambia

**Table 2:** Recommendations by national nutritional care and support guidelines for non-pregnant HIV-infected adults

Component of nutritional care and support	Does the guideline specify…	Countries providing guidance	Countries *not* providing guidance
*Assessment of nutritional status*	… that an assessment of nutritional status should be carried out at least once?	Botswana, Kenya, Rwanda, South Africa, Swaziland, Tanzania, Zambia	Uganda
	… when in the HIV care cascade nutritional status should first be assessed?	Botswana, Kenya, South Africa	Rwanda, Swaziland, Tanzania, Uganda, Zambia
	… how regularly nutritional status should be re-assessed?	Rwanda, Kenya, South Africa	Botswana, Swaziland, Tanzania, Uganda, Zambia
	… how the assessment should be carried out?	Botswana, Kenya, Rwanda, South Africa, Swaziland, Tanzania, Zambia	Uganda
*Dietary counseling*	… that dietary counseling should be provided?	Botswana, Kenya, Rwanda, South Africa, Swaziland, Tanzania, Uganda, Zambia	-
	… when in the HIV care cascade it should first be provided?	-	Botswana, Kenya, Rwanda, South Africa, Swaziland, Tanzania, Uganda, Zambia
	… how regularly it should be provided?	-	Botswana, Kenya, Rwanda, South Africa, Swaziland, Tanzania, Uganda, Zambia
	… what topics should be covered during the counseling?	Botswana, Kenya Rwanda, Swaziland, Tanzania, Uganda, Zambia	South Africa, Uganda
*Micronutrient supplementation*	… whether a micronutrient supplement should be provided?	Kenya, Zambia	Botswana, Rwanda, South Africa, Swaziland, Tanzania, Uganda
	… under what circumstances a micronutrient supplement should be provided?	Kenya, Zambia	Botswana, Rwanda, South Africa, Swaziland, Tanzania, Uganda
	… which micronutrient(s) should be provided?	Kenya	Botswana, Rwanda, South Africa, Swaziland, Tanzania, Uganda, Zambia
	… the dosage of at least one micronutrient supplement?	-	Botswana, Kenya, Rwanda, South Africa, Swaziland, Tanzania, Uganda, Zambia
*Ready-to-use therapeutic and supplementary foods (RUTFs)*	… whether a RUTF should be provided?	Kenya, Zambia	Botswana, Rwanda, South Africa, Swaziland, Tanzania, Uganda
	… under what circumstances a RUTF should be provided?	Kenya, Zambia	Botswana, Rwanda, South Africa, Swaziland, Tanzania, Uganda
	… which RUTF(s) should be provided?	-	Botswana, Kenya, Rwanda, South Africa, Swaziland, Tanzania, Uganda, Zambia
	… the dosage of at least one RUTF?	Kenya, Zambia	Botswana, Rwanda, South Africa, Swaziland, Tanzania, Uganda
*Food subsidies*	… whether a food subsidy should be provided?	-	Botswana, Kenya, Rwanda, South Africa, Swaziland, Tanzania, Uganda, Zambia
	… under what circumstances a food subsidy should be provided?	-	Botswana, Kenya, Rwanda, South Africa, Swaziland, Tanzania, Uganda, Zambia
	… how the food subsidy should be provided (e.g., vouchers, food baskets, etc.)?	-	Botswana, Kenya, Rwanda, South Africa, Swaziland, Tanzania, Uganda, Zambia

*Abbreviations*: *HIV* human immunodeficiency virus, *RUTF* Ready-to-use therapeutic or supplementary food

## Discussion

### The current evidence base on nutritional care and support for adults living with HIV

**Fig. 1:**
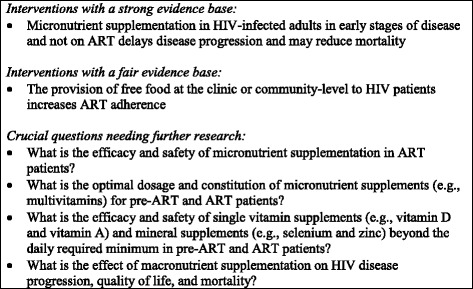
Summary of the evidence base on nutritional care and support for adults living with HIV

Nutritional care and support interventions can affect HIV disease progression directly through physiological pathways, and indirectly through effects on other components of HIV care, such as retention in care and ART adherence. Figure 1 summarizes our assessment of the body of evidence on nutritional care and support for adults living with HIV.

#### Dietary counseling

A 2015 systematic review of dietary counseling and nutritional care and support interventions (excluding micronutrient supplementation) in HIV-infected patients identified only one study, which investigated the effects of dietary counseling in isolation [38]. The outcomes assessed in the randomized trial of 84 adult ART patients in Nigeria were serum hemoglobin concentration and body mass index (BMI) [39]. After the six months follow-up period, the group who received monthly dietary counseling at its routine ART visits had a higher average hemoglobin concentration (12.1 versus 11.2 mg/dL, *p* = 0.0015) than the group who did not. The effect of dietary counseling on BMI was only significant among female participants (24.9 kg/m^2^ versus 21.8 kg/m^2^, *p* = 0.0005) but not in males nor in the study group as a whole.

#### Micronutrient supplementation

To date, three randomized double-blind placebo-controlled trials have been conducted to investigate the effect of a multiple micronutrient supplement on HIV disease progression in patients not on ART [40]. These trials are summarized in Table 3. While their results vary somewhat by endpoint, likely due to different endpoint definitions and varying statistical power, they are consistent in reporting a beneficial effect of multiple micronutrient supplementation on HIV disease progression and/or mortality. Both the trial in Botswana and Tanzania found a beneficial effect on disease progression while the Thailand study, in which participants were on average in a more advanced disease stage than in the other two trials, found no effect [41–43]. The Tanzania and Thailand studies also report data on mortality with both trials finding a lower risk of HIV-related death in the multiple micronutrient arm(s). The difference was marginally significant in the Tanzania trial comparing those assigned to the multivitamin (without vitamin A) to the placebo group (relative risk of 0.73 with a 95 % confidence interval (CI) of 0.51–1.04; *p* = 0.09). In the Thailand study, the effect on mortality was significant among those with a CD4-cell count less than 100 cells/μL (hazard ratio of 0.26 with a 95 % CI of 0.07–0.97; *p* = 0.03) and marginally significant for those with a CD4-cell count less than 200 cells/μL (hazard ratio of 0.37 with a 95 % CI of 0.13–1.06; *p* = 0.05).

**Table 3:** The impact of multiple micronutrient supplementation^a^ in HIV-infected adults not on ART on HIV disease progression and mortality – a summary of randomized double-blind placebo-controlled trials

Reference	Country	Follow-up period	Study population	Intervention	Sample size		Endpoint *(disease progression)*	Result	Endpoint *(mortality)*	Result
					*Intervention*	*Placebo*				
Baum et al. 2013 [41]	Botswana	24 months	HIV+ adults not on ART with CD4 > 350 cells/μL	Arm 1: Multivitamin^b^ + selenium^c^; Arm 2: Multivitamin^b^; Arm 3: Selenium^c^	Arm 1: 220; Arm 2: 219; Arm 3: 216	217	CD4-cell count falling to ≤250 cells/μL	*Hazard ratio* ^*d*^ *(95 % CI):* Arm 1 vs placebo: 0.46 (0.25–0.85)^*d*^; Arm 2 vs placebo: 0.83 (0.48–1.42)^*d*^; Arm 3 vs placebo: 0.76 (0.44–1.32)^*d*^	Not assessed	Not assessed
Fawzi et al. 2004 [42]	Tanzania	60 / 71 months^e^	HIV+ pregnant^f^ women not on ART	Arm 1: Multivitamin^g^ with vitamin A^h^; Arm 2: Multivitamin^g^ without vitamin A; Arm 3: Vitamin A^h^	Arm 1: 268; Arm 2: 271; Arm 3: 272	267	≥2-stage increase in WHO clinical stage^i^	*Relative risk (95 % CI):* Arm 1 vs placebo: 0.74 (0.59–0.94); Arm 2 vs placebo: 0.66 (0.52–0.84); Arm 3 vs placebo: 0.74 (0.58–0.93)	Progression to clinical stage 4 or death from AIDS-related causes^j^	*Relative risk (95 % CI):* Arm 1 vs placebo: 0.80 (0.58–1.10); Arm 2 vs placebo: 0.71 (0.51–0.98); Arm 3 vs placebo: 0.88 (0.64–1.19)
									Death from HIV-related causes	*Relative risk (95 % CI):* Arm 1 vs placebo: 0.91 (0.64–1.28); Arm 2 vs placebo: 0.73 (0.51–1.04); Arm 3 vs placebo: 0.93 (0.66–1.32)
Jiamton et al. 2003 [43]	Thailand	11 months	HIV+ adults not on ART^k^ with a CD4-cell count between 50 and 550 cells/μL	Multiple micronutrient supplement^l^	242	239	Median CD4-cell count ^m^	I: 200 cells/μL (IQR: 66–358) vs C: 232 cells/μL (IQR: 73–377); *p* > 0.3^n^	Death from HIV-related causes	*Hazard ratio* ^*d*^ *(95 % CI):* 0.53 (0.22–1.25)^o^
							Mean plasma viral load^m^	I: 4.4 log10 copies per ml (95 % CI: 4.1–4.7) vs C: 4.5 log10 copies per ml (95 % CI: 4.3–4.8); *p* = 0.4		

*Abbreviations*: *HIV* human immunodeficiency virus, *HIV+* HIV-infected, *ART* antiretroviral therapy, *CD4* cluster of differentiation 4, *CI* confidence interval, *vs* versus, *WHO* World Health Organization, *I* intervention arm, *C* control arm

^a^A multiple micronutrient supplement was defined as a supplement containing at least five different micronutrients

^b^The multivitamin consisted of a daily supplement containing vitamin B1 (20mg), vitamin B2 (20mg), vitamin B6 (25mg), niacin (100mg), vitamin B12 (50μg), vitamin C (500mg), vitamin E (30mg), and folic acid (0.8mg)

^c^The dose of selenium was 200μg per day

^d^adjusted for age, sex, baseline CD4-cell count, baseline HIV viral load, and baseline BMI

^e^This is the median follow-up time, which was 60 months (interquartile range of 14 to 79 months) for stage of HIV disease and 71 months (interquartile range of 46 to 80 months) for survival

^f^Only a small proportion of follow-up time (~6 %) was during pregnancy

^g^The multivitamin consisted of a daily supplement containing vitamin B1 (20mg), vitamin B2 (20mg), vitamin B6 (25mg), niacin (100mg), vitamin B12 (60μg), vitamin C (500mg), vitamin E (30mg), and folic acid (0.8mg)

^h^The vitamin A supplementation consisted of a daily supplement containing vitamin A (5,000IU) and beta-carotene (30mg)

^i^The primary endpoint of the trial was progression to WHO clinical stage 4 or AIDS-related death

^j^This is the primary endpoint of the trial

^k^Some participants may have had access to ART from pharmacies

^l^The multiple micronutrient supplement consisted of two tablets per day, together containing vitamin A (3000μg), beta-carotene (6mg), vitamin D3 (20μg), vitamin E (80 mg), vitamin K (180μg), vitamin C (400mg), vitamin B1 (24mg), vitamin B2 (15mg), vitamin B6 (40mg), vitamin B12 (30μg), folacin (100μg), panthothenic acid (40mg), iron (10mg), magnesium (200mg), manganese (8mg), zinc (30mg), iodine (300μg), copper (3mg), selenium (400μg), chromium (150μg), and cysteine (66mg)

^m^The primary endpoint of the trial was HIV-related mortality

^n^The exact p-value was not reported

^o^Among participants with a CD4-cell count <100 cells/μL at enrolment, the relative risk ratio was statistically significant at 0.26 (95 % CI: 0.07–0.97)

In addition to the trials shown in Table 3, a recent meta-analysis on the subject also included observational studies and randomized trials that were not carried out exclusively in HIV-infected participants (but which reported data disaggregated by HIV-status) [40]. It identified one relevant observational study and seven randomized trials, six of which were carried out in sub-Saharan Africa (three in Tanzania) [40]. Synthesizing data from three trials (2,249 participants), this Bayesian meta-analysis estimated a relative risk of 0.62 (95 % confidence interval: 0.37–0.96) of HIV disease progression in subjects assigned to receiving the multiple micronutrient supplement versus controls. Pooling data from seven studies (4,095 participants), the effect on mortality, however, was not statistically significant (relative risk of 0.84 with a 95 % confidence interval of 0.38 to 1.85).

Although there is substantial evidence for beneficial effects of multiple micronutrient supplements, including multivitamins, on disease progression in patients in early stages of disease and *not* on ART, it is unclear whether this effect also applies to ART patients. To date, relatively few and small trials of single or multiple micronutrient supplements in ART patients have published their results, with some suggesting a beneficial impact [44–48] whereas others finding no significant effect [49, 50]. Enrolling patients who initiated ART less than six months ago, a randomized double-blind placebo-controlled trial in Uganda investigated the effect of a multivitamin supplement on immune reconstitution, weight gain, and quality of life of ART patients [51]. The trial found no significant impact from the multivitamin on any of its three primary endpoints [52]. In addition, there is evidence of possible harm from high-dose multivitamin supplements in patients on ART with a randomized trial, comparing standard-dose to high-dose multivitamin supplementation in Dar es Salaam, having been stopped early due to an increased risk of raised alanine transaminase levels in the high-dose multivitamin group [53].

An additional gap in the evidence base on micronutrients and HIV is the effect of *single* micronutrient supplements in pre-ART and ART patients. In particular, there is promising evidence from observational studies in ART patients that vitamin D supplementation may delay HIV disease progression [54, 55], reduce the incidence of pulmonary tuberculosis, opportunistic infections, and wasting [56], and, in conjunction with calcium, reduce bone loss associated with some antiretroviral drugs [57]. Providing further encouraging evidence, a recent phase 1 clinical trial found that vitamin D supplementation had more pronounced beneficial effects on the immune system of HIV-infected than HIV-uninfected adults [58]. Several randomized trials are currently ongoing to study the efficacy and safety of vitamin D supplementation in ART patients. At the moment, however, the evidence base for the effect of single micronutrient supplements, including minerals (e.g., selenium or zinc), on HIV morbidity and mortality in adults is still weak; a conclusion also reached by a 2010 Cochrane review on micronutrient supplementation in children and adults with HIV infection [59].

#### Macronutrient supplementation including ready-to-use therapeutic and supplementary foods

Both quantitative and qualitative studies have shown food insecurity to be a critical barrier to ART adherence and retention in HIV care among adults living with HIV [60]. Somewhat unsurprisingly, therefore, a recent systematic review reported that five of the seven observational studies it identified on the subject found that food provision at the clinic- or community-level improved ART adherence [38, 60]. It, however, did not find a randomized trial on this question.

A recent Cochrane review evaluated the effectiveness of oral macronutrient supplementation on morbidity and mortality in adults and children living with HIV [61]. The review identified 14 randomized trials, of which ten trials (1,725 participants) were conducted in adults. Only three of these ten trials took place in a low-or middle-income country, namely Kenya [62], the Central African Republic [63], and India [64]. The review concluded that dietary counseling along with macronutrient supplementation (fortified with micronutrients) significantly increased energy and protein intake compared to dietary counseling only. There is, however, insufficient evidence regarding the impact of macronutrient supplementation on anthropometric and clinical outcomes, including mortality. Yet, it is fair to assume that malnourished individuals, including adults with a BMI <18.5 kg/m^2^, need to be supported with an optimal quantity and quality of nourishment as part of standard clinical practice. 

### Implications of the findings

We found that governments in sub-Saharan Africa do not generally include specific recommendations or guidance on nutritional care and support for adults living with HIV in their national HIV treatment guidelines. Yet, as discussed in the previous section, substantial evidence exists for the beneficial effect and safety of certain nutritional care and support interventions for adults living with HIV. It is our view that, at a minimum, the WHO’s HIV treatment guidelines should include a strong recommendation for the use of daily multiple micronutrient supplements (that include the recommended daily allowance for these nutrients) for HIV-infected adults not (yet) on ART, coupled with regular dietary counseling on the use of nutritionally dense animal and plant-sourced foods. Furthermore, guidelines should emphasize the importance of regularly assessing patients’ BMI to detect under-nourishment. The management of undernourished patients should largely be based on locally available foods where possible, but would ideally also include ready-to-use therapeutic foods for a limited time until patients have regained their optimal nutritional status.

Generalizing lessons from this study to other clinical areas is problematic given that this is a case study rather than a comprehensive assessment across clinical areas. Nonetheless, this study provides evidence for two hypotheses: 1) if the WHO does not issue recommendations on a certain clinical question, then countries in sub-Saharan Africa will not provide specific guidance on this question to their clinicians, and 2) this is the case even if substantial evidence exists for a specific recommendation on the question. Even if only 1) is true but 2) is false, this situation will be undesirable because clinicians are less well equipped than the WHO or Ministries of Health to systematically identify, assess, and summarize existing evidence, and to access the opinion of experts in the field. Yet, clinicians will inevitably have to make decisions on these questions. For example, nutritional supplements, such as multivitamins, are readily available to many patients in sub-Saharan Africa from formal or informal pharmacies and drug sellers [65]. The subject of nutrition and nutritional supplements in relation to HIV has also received substantial media coverage in many sub-Saharan African countries due to the AIDS denialism movement in South Africa [66]. It is, thus, likely that some HIV patients enquire from clinicians whether they should take a nutritional supplement, and if so, which one and at what dose.

If the first or both of our hypotheses outlined above are found to be true, such as through a comprehensive assessment across clinical areas, then we would recommend two approaches for the WHO’s clinical guidelines: 1) providing more specific guidance (which may have to be largely expert-driven in areas where the evidence base is weak or conflicting), and/or 2) clearly outlining questions and areas for which the WHO is unable to provide specific guidance, but which should be addressed in national guidelines.

### Limitations

A limitation of this study is its restriction to countries in sub-Saharan Africa, in which English is an official language. To the best of our knowledge, there is, however, no particular reason why the fairly consistent pattern of our findings across countries would have been different if we had also included countries in sub-Saharan Africa, in which English is not one of the official languages. A second limitation is that we relied on the identification of guidelines through online searches. As it is unusual to not publish national clinical guidelines online, we are confident that we identified the latest relevant guideline for the majority of the included countries.

## Conclusions

This case study suggests that despite substantial evidence for some nutritional interventions, such as multiple micronutrient supplementation for pre-ART patients, countries in sub-Saharan Africa are looking to the WHO’s guidelines for guidance in deciding what recommendations to include in their national clinical guidelines. In the case of nutritional care and support, the onus to make a decision is currently implicitly left to clinicians in sub-Saharan African countries who are arguably less well equipped to systematically evaluate the existing evidence and have less access to expert opinion than WHO or national governments. If this tendency of a lack of guidance by WHO resulting in a lack of clinical guidance at the national level is found to be true across other clinical areas, then this has important practical implications for WHO and its guidelines in general. In this case, we would suggest that WHO provides specific recommendations (including expert-driven guidance for questions with a lacking evidence base) in areas where clinical decisions are inevitably being made during patient care, and/or highlights clinical questions for which it is unable to provide specific guidance but which WHO feels ought to be addressed in national guidelines.
